# Noncovalent interactions and odd–even effect control the high-pressure crystal structures of phenyl alcohols

**DOI:** 10.1107/S2052252526009504

**Published:** 2026-11-05

**Authors:** Joanna Grelska, Roman Gajda, Joanna Krzeszczakowska, Changyong Park, Krzysztof Woźniak, Wojciech A. Sławiński

**Affiliations:** a University of Warsaw Faculty of Chemistry Pasteura 1 02-093Warsaw Poland; b High Pressure Collaborative Access Team (HPCAT), X-ray Science Division Argonne National Laboratory Lemont Illinois60439 USA; Stellenbosch University, South Africa

**Keywords:** phenyl alcohols, intermolecular interactions, crystallization and crystal growth, crystallization under non-ambient conditions, hydrogen bonding, density functional theory, molecular crystals

## Abstract

We report four high-pressure crystal structures of a homologous series of phenyl alcohols starting with benzyl alcohol, C_6_H_5_(CH_2_)_*n*_OH (*n* = 1–4). Results were obtained using synchrotron and laboratory single-crystal X-ray diffraction supported by powder diffraction and periodic density functional theory calculations. For three alcohols, 2-phenylethanol, 3-phenyl-1-propanol and 4-phenyl-1-butanol, these represent the first crystal structures of the pure compounds. All structures are characterized by hydrogen-bonded chains of O—H⋯O interactions, complemented by stabilizing C—H⋯π and C—H⋯O contacts. Despite similarities in local bonding motifs, distinct packing arrangements emerge depending on molecular chain length. Even alcohols (*n* = 2, 4) adopt closely related layered structures with efficient packing, whereas odd analogs (*n* = 1, 3) exhibit less dense and more asymmetric arrangements. High pressure induces crystallization under 1 GPa, highlighting the role of pressure in accessing otherwise undiscovered crystal forms.

## Abstract 2

We report the structures of four alcohols from a homologous series: benzyl alcohol, 2-phenylethanol, 3-phenyl-1-propanol and 4-phenyl-1-butanol, obtained under high pressure (1 GPa). Their structures were determined using single-crystal X-ray diffraction supported by powder diffraction and periodic density functional theory calculations. The even alcohols are more efficiently packed than their odd counterparts, and all of these structures are characterized by strong hydrogen bonds and stabilizing aromatic interactions.

## 1. Introduction

Hydrogen bonding and aromatic π-interactions are present in the crystal structures of many chemically relevant materials. One of the most prominent examples is DNA, where nucleobase stabilization arises from π-stacking, while base pairs bind through directional hydrogen bonds (Chattopadhyaya *et al.*, 1988 ▸; Watson & Crick, 1953 ▸). These non­covalent interactions have been widely studied (Katrusiak, 2026 ▸; Nayak *et al.*, 2010 ▸; Steiner, 2002 ▸; Desiraju, 1991 ▸; Gajda *et al.*, 2019 ▸; Katrusiak *et al.*, 2010 ▸; Budzianowski & Katrusiak, 2006 ▸; Kirchner *et al.*, 2011 ▸; Podsiadło *et al.*, 2010 ▸; Thakur *et al.*, 2011 ▸; Marciniak *et al.*, 2016 ▸; Prus *et al.*, 2022 ▸; Moorthy *et al.*, 2012 ▸; Desiraju, 1995 ▸). It has been shown that hydrogen bonds typically organize into chain or dimer motifs, while aromatic rings preferentially adopt slipped-stack or T-shaped arrangements rather than ideal face-to-face stacking. Substitution on the aromatic ring or modification of the hydroxyl group not only strongly influences packing efficiency and conformational preferences but also affects thermodynamic stability and macroscopic properties. Crystallographic studies have demonstrated that even small structural modifications, for example in benzyl derivatives the substitution of functional groups such as hydroxyl, sulfhydryl, amino, cyano, chloro or bromo, can significantly alter the molecular packing scheme (Nayak *et al.*, 2010 ▸). Understanding the coexistence between hydrogen bonding and aromatic interactions in crystal structures provides a benchmark for predicting the crystal forms of new functional materials.

Phenyl alcohols represent an important class of organic compounds in which both the hydroxyl group and the aromatic ring act as sources of intermolecular interactions. Their structural behavior is governed by the interplay between classical hydrogen bonding and aromatic π-interactions, making them valuable model systems for investigating competing noncovalent forces in condensed phases. The coexistence of these interactions often leads to complex intermolecular arrangements and structural disorder. It also may be the reason why many phenyl alcohols do not crystallize easily upon cooling and instead form glassy states. Consequently, experimental information about their crystalline structures remains limited. However, recent advances in high-pressure diffraction techniques (Katrusiak, 2026 ▸; Sacharczuk *et al.*, 2026 ▸) have made it possible to obtain crystalline phases that are inaccessible under ambient conditions. By examining a series of phenyl alcohols that differ only slightly in molecular structure, one obtains a control group for probing how multiple noncovalent interactions shape crystal packing and stability.

This systematic study analyzes model phenyl compounds belonging to a homologous series. In these simple organic molecules, the aromatic ring and the hydroxyl group are connected by an alkyl side chain whose length varies in the number of carbon atoms. The distance between these functional groups is small enough to prevent any intramolecular interactions. The general formula of the series is C_6_H_5_(CH_2_)_*n*_OH, where *n* = 1 (benzyl alcohol, **BA**), *n* = 2 (2-phenylethanol, **2P**), *n* = 3 (3-phenyl-1-propanol, **3P**) or *n* = 4 (4-phenyl-1-butanol, **4P**). All the compounds are liquids at ambient conditions. Upon cooling, only **BA** crystallizes, whereas the other compounds (**2P**, **3P** and **4P**) vitrify, forming glassy states with glass-transition temperatures of 184, 182 and 181 K, respectively (Soszka *et al.*, 2021 ▸). Here we report four new high-pressure crystal structures and analyze them in terms of molecular conformations and intermolecular interactions. In the investigated series similarities in crystal packing were observed, as well as resemblances to other crystal structures of simple aliphatic alcohols described in the literature.

## 2. Results and discussion

### 2.1. High-pressure crystal structures of phenyl alcohols

High-pressure crystal structures of this phenyl alcohol series were determined using synchrotron radiation (SR-SCXD) and laboratory (SCXD) single-crystal X-ray diffraction. The crystal structures of four alcohols at their crystallization point with basic crystallographic parameters are summarized in Table 1 ▸, with full details provided in the supporting information. For three of the alcohols, **BA**, **2P** and **3P**, a second pressure point around 1.5 GPa was achieved with no phase transition observed (details are provided in the supporting information). The total of seven crystal structures reported in this study have been deposited at the Cambridge Crystallographic Data Centre (CCDC), under deposition numbers CCDC 2587070–2587076.

**Figure 1:**
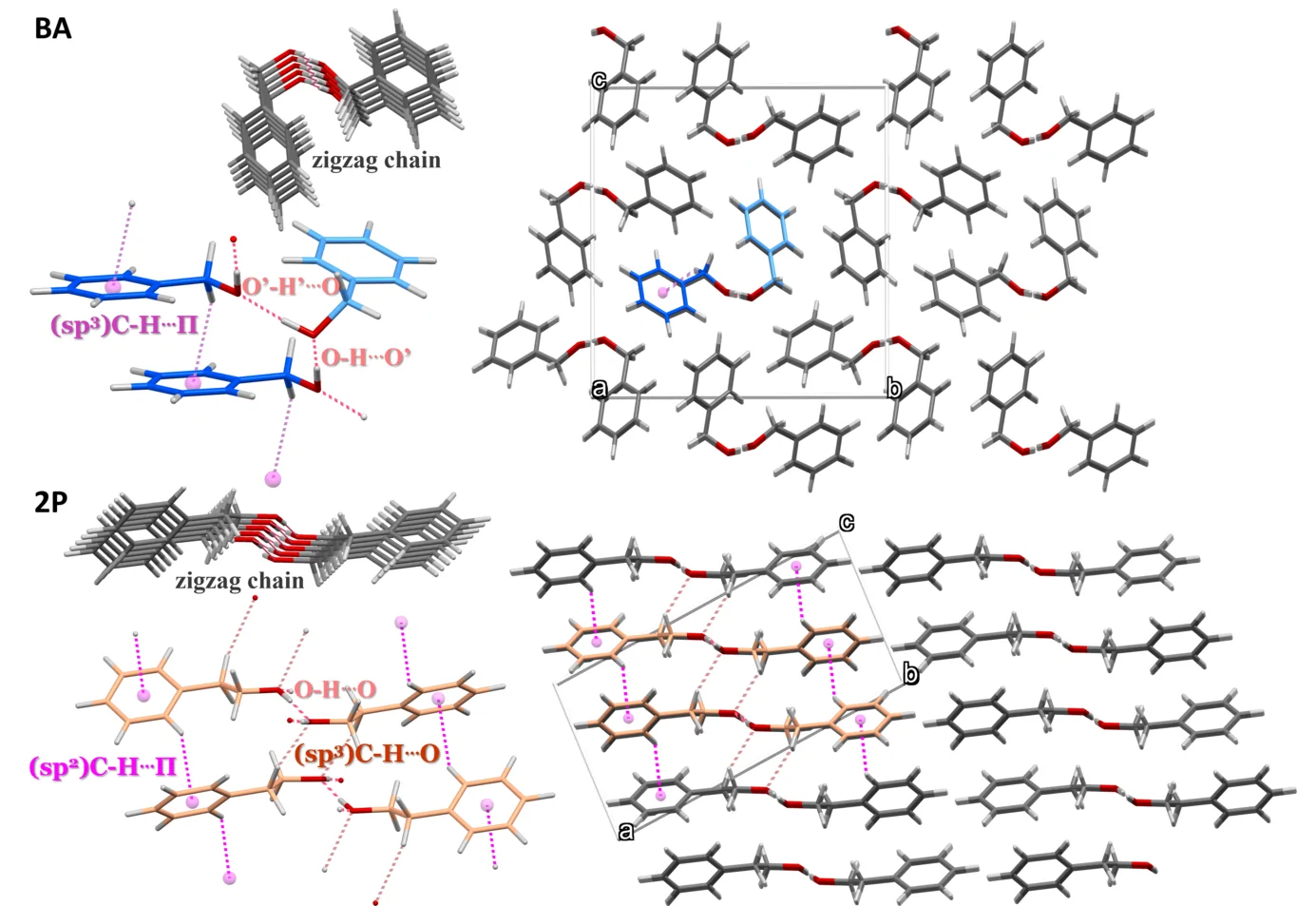
Projections of crystal structures and selected molecules for **BA** and **2P**, with dimer interactions marked.

*Benzyl alcohol*. The first in the investigated homologous series of alcohols is **BA**, also known as phenylmethanol. **BA** is the only compound in the series with a known low-temperature crystal structure (Nayak *et al.*, 2010 ▸). It crystallizes below 210 K in the monoclinic crystal system with space group *P*2_1_. A characteristic feature of this structure is hydrogen-bonded chains of molecules formed through O—H⋯O contacts. In the low-temperature structure of **BA** there are no interactions involving aromatic protons. In turn, methyl carbon interactions with the aromatic ring (*sp*^3^)C—H⋯π are present and these contacts stabilize hydrogen-bonded chains of molecules (Nayak *et al.*, 2010 ▸). The analogous interactions can be found in the high-pressure structure reported here, although characterized by a different space group and unit cell. We revealed that at high pressure, **BA** crystallizes in the orthorhombic crystal system with space group *P*2_1_2_1_2_1_. In this structure, two independent molecules are connected alternately by hydrogen bonds, creating zigzag chains along the *a* axis (see Fig. 1 ▸).

*2-Phenylethanol*. According to our knowledge neither the low-temperature nor the high-pressure structure of **2P** has been deposited in the Cambridge Structural Database (CSD) (Groom *et al.*, 2016 ▸) before. However, there are deposited structures of solvates of **2P** with other compounds (Galloway *et al.*, 2012 ▸; Ou *et al.*, 2022 ▸; Wenz *et al.*, 2017 ▸; Kajiki *et al.*, 2016 ▸). We report for the first time the structure of pure **2P** obtained under a pressure of 0.72 GPa. This structure clearly differs from the one detected for **BA** (see Fig. 1 ▸). The **2P** compound crystallizes in space group *P*2_1_/*c* where molecules are organized in rows, and within a row molecules are connected by hydrogen bonds forming zigzag chains along the *b* axis.

**Figure 2:**
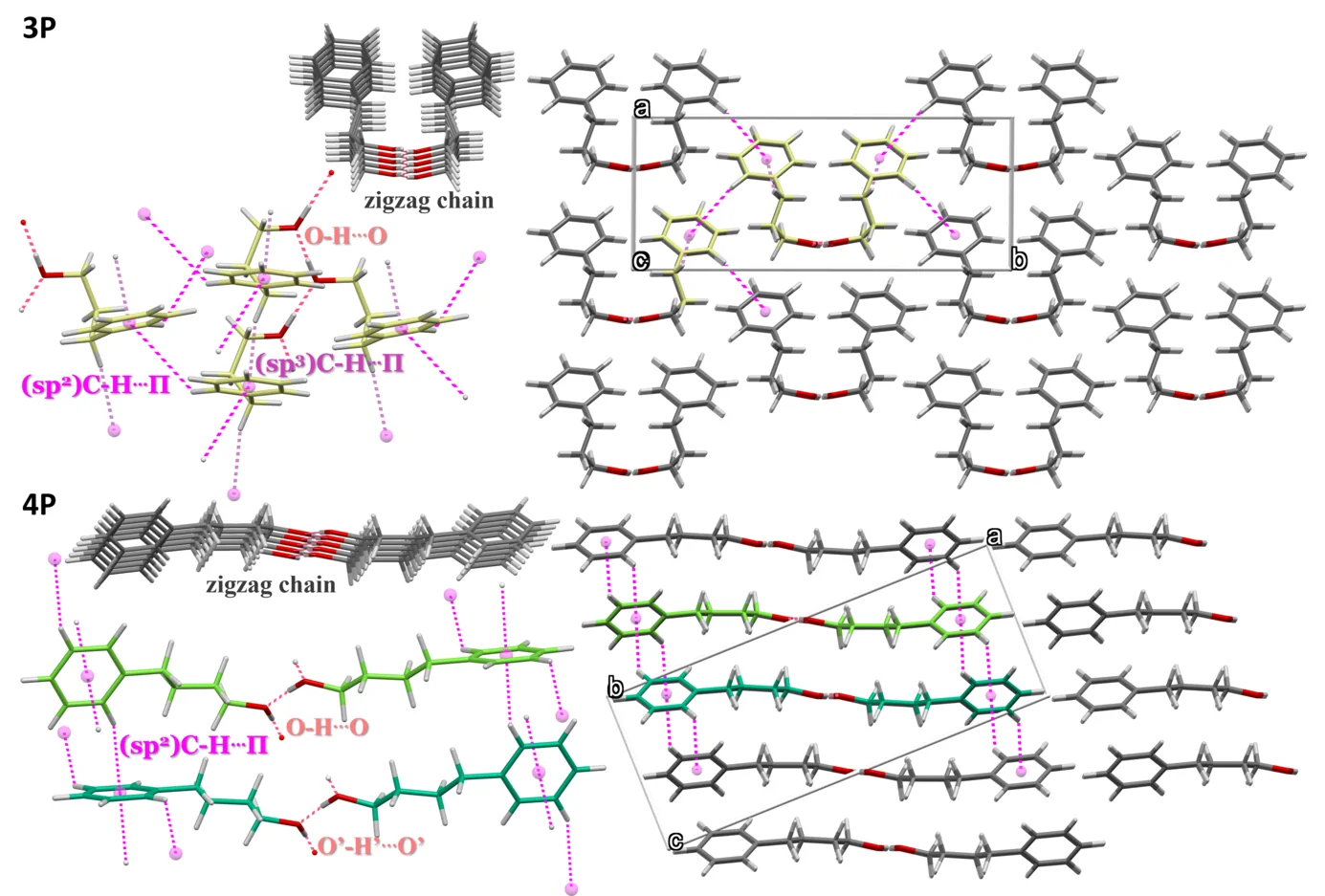
Projections of crystal structures and selected molecules for **3P** and **4P** with dimer interactions marked.

*3-Phenyl-1-propanol*. In the case of **3P**, there are many organometallic compounds in the CSD in which this molecule is coordinated to bigger organometallic moieties containing osmium or ruthenium (Miyaki *et al.*, 2000 ▸; González-Fernández *et al.*, 2019 ▸; Guo *et al.*, 2021 ▸; Infante-Tadeo *et al.*, 2021 ▸). We have determined for the first time the structure of pure **3P** under a pressure of 0.66 GPa. It is worth noting that the sample does not crystallize immediately, but does so after approximately 30 minutes at a pressure of about 1 GPa. At ∼2 GPa, the sample does not crystallize at all even after an overnight incubation; instead, it most likely transitions into a glassy state. The **3P** alcohol crystallizes in space group *Cc* with a large β angle, Fig. 2 ▸. Molecules are hydrogen-bonded, creating zigzag chains along the *c* axis. Interestingly, all chain-bonded molecules have the same orientation head-to-tail along the *a* axis.

*4-Phenyl-1-butanol*. Among the phenyl alcohols that were studied, **4P** was the hardest to grow as a single crystal. In the CSD, we have found only one crystal structure, where **4P** is coordinated to ruthenium (Tian *et al.*, 2018 ▸). We report for the first time the crystal structure of pure **4P**. In the structure, long molecules arranged next to each other create extended layers, which probably inhibit the easy growth of subsequent pairs of molecules along the *a* axis. There is a clear resemblance to the structure of **2P**. In both structures, two hydrogen-bonded molecules are oriented in the direction of the long molecular axis, thus forming zigzags along the *b* axis (see Fig. 2 ▸). In **4P** there are two independent molecules that alternate in every other zigzag. However, the experimental data for this compound are of low quality, so we cannot exclude the possibility that the **4P** structure could be even more complicated.

### 2.2. Powder diffraction evidence for layered crystal packing

**Figure 3:**
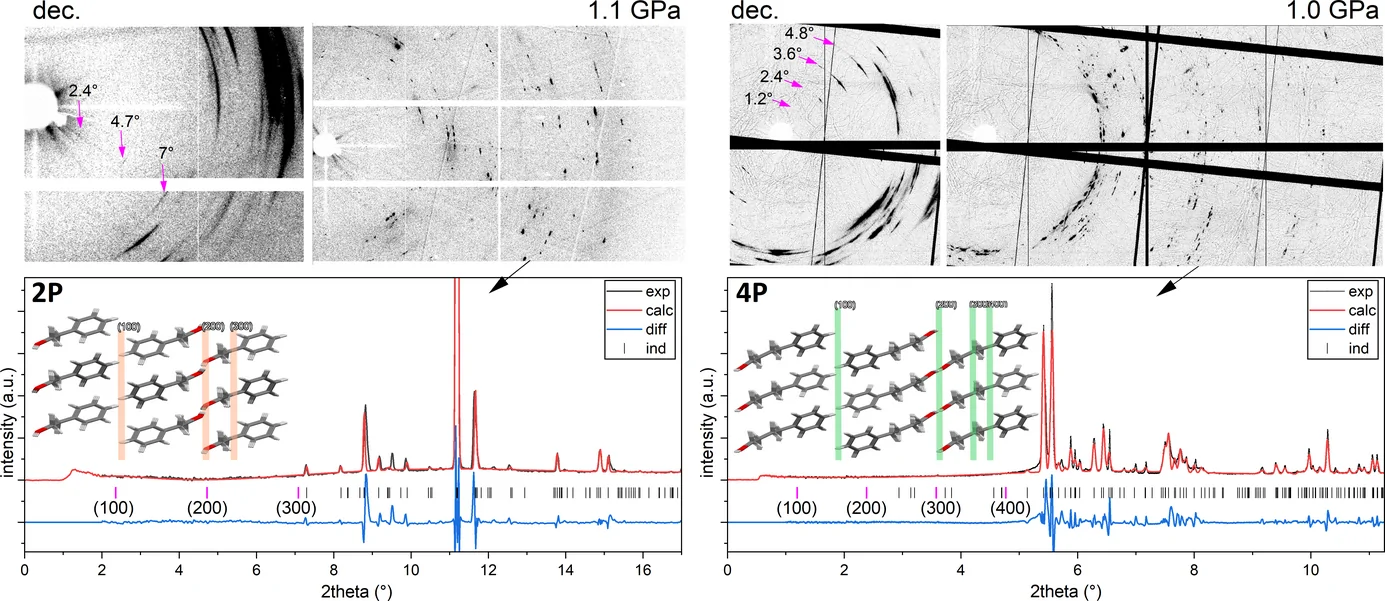
(Top) Powder diffraction patterns for **2P** (left) and **4P** (right) measured at the pressure point and on decompression (dec.). (Bottom) Powder diffraction Le Bail refinements, with the crystal planes seen on decompression images marked. The insets show projections of the crystal structures used to visualize an (*h*00) plane family.

High-pressure synchrotron radiation powder X-ray diffraction experiments (SR-PXD) were conducted for **2P** and **4P**. The two-dimensional powder patterns presented in Fig. 3 ▸ show that, at a pressure around 1 GPa, both alcohols exhibit a polycrystalline texture with high preferred orientation. Hence only Le Bail fitting was used, which successfully confirmed the structures obtained based on the SCXD measurements. The powder patterns measured on decompression indicate a more homogeneous polycrystalline form, albeit with a disordered and deformed structure. Owing to significant peak broadening and the incomplete powder sampling, structural refinement could not be performed. The raw integrated powder patterns obtained on decompression are displayed in the supporting information.

The data measured on decompression show an important feature not seen at 1 GPa: characteristic Bragg peaks at low scattering angles. It is evident that in **2P** the reflections at 2.4, 4.7 and 7° seen in the decompression image correspond to crystallographic planes (100), (200) and (300), respectively. In **4P** Bragg peaks seen on decompression at 1.2, 2.4, 3.6 and 4.8° correspond to crystal planes (100), (200), (300) and (400), respectively. Fig. 3 ▸ shows 2D diffraction patterns as well as integrated 1D plots for **2P** and **4P** with characteristic Bragg peaks marked. These peaks are a fingerprint of crystal planes aligned along the molecules’ longest axes, highlighted in insets in Fig. 3 ▸.

Simulated powder diffraction patterns for all phenyl compounds are presented in the supporting information. Notably, the Bragg peaks at low diffraction angles are characteristic only for **2P** and **4P** among the alcohols studied in the series. In comparison, the odd alcohols lack the long molecular axis arrangement. This clear evidence of layer packing of the even alcohols underlines the molecular alkyl chain length as an important factor in the organization of molecules in crystal structure.

### 2.3. Energetic hierarchy of layer, lattice and dimer interactions

**Figure 4:**
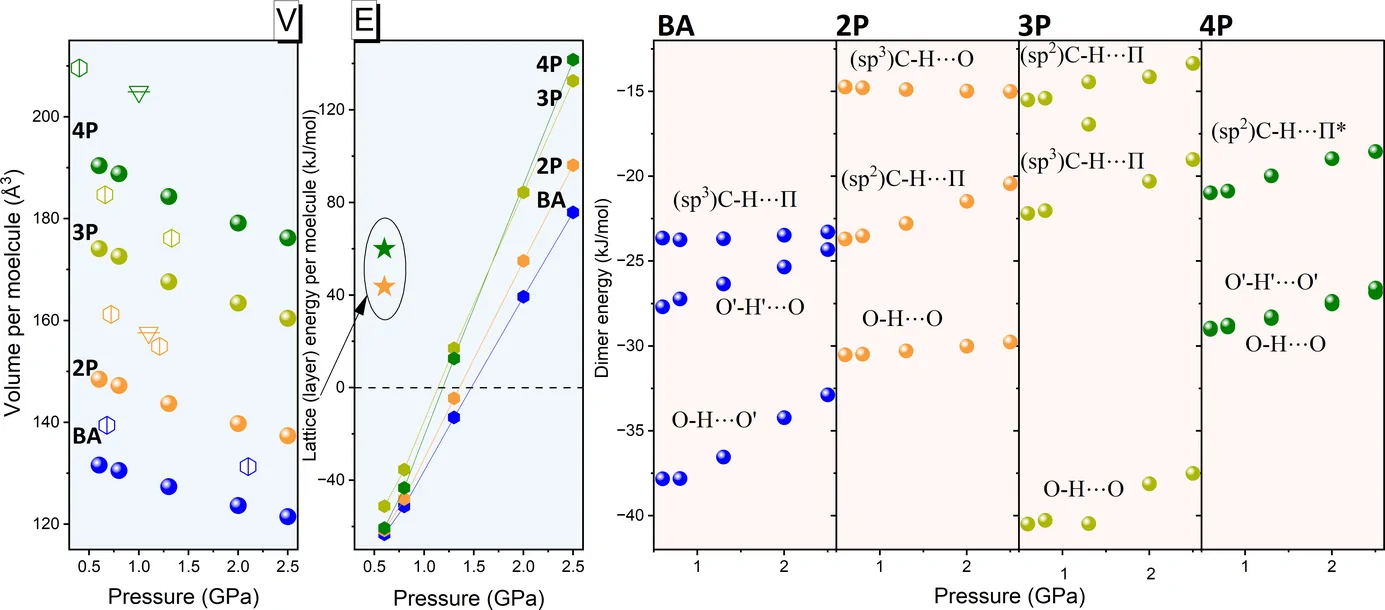
On the left, the volume per molecule obtained from DFT calculated models (solid, colored dots), experimentally obtained from SR-SCXD and SCXD (open circles with vertical lines) and SR-PXD (open triangles with horizontal lines), along with model lattice energies (solid, colored dots) and layer energies (colored stars). On the right, dimer energies calculated for optimized models, with assigned interaction types.

Lattice energies, volumes and dimer energies, presented in Fig. 4 ▸, were calculated using periodic density functional theory (DFT) based on a series of optimizations performed at different pressures. The precise values are tabled in the supporting information. Interestingly, the lattice energy values are negative only at low pressures, up to ∼1.3 GPa. At higher pressures, the lattice energy becomes higher and unfavorable to bind a molecule in the system. Notably, at the lowest pressure point (0.6 GPa), the lattice energy (−40 kJ mol^−1^) is only slightly negative compared with the calculated dimer energies. This can be explained by the coexistence of interactions with positive energies balanced by attractive forces. Additionally, for **2P** and **4P**, attachment energies of the (100) layer were calculated (see Fig. 3 ▸ and the supporting information for visualization). The values of energies between the layers (100) are significantly positive (see the star symbols in Fig. 4 ▸), showing that contacts of aromatic rings between the layers are repulsive. That is, the crystal structures must be stabilized by other attractive forces.

Three lowest energies of attractive interactions between dimers, which were found for each alcohol structure, are shown in Fig. 4 ▸. In every structure there are many such dimer equivalents, the interaction types of which are assigned and visualized in Fig. 1 ▸ and Fig. 2 ▸. Tables containing dimer energies, as well as interaction distances and angles, are provided in the supporting information. In all the alcohol crystal structures, the O—H⋯O hydrogen bond exhibits the lowest energy, hence it is the strongest dimer interaction in the system, with the lowest energy values observed for **BA** and **3P** compared with the other alcohols. All dimer energies increase with increasing pressure, whereas interaction distances decrease due to higher density (see the supporting information). In **BA**, two independent molecules form hydrogen bonds with different energies (−27 and −37 kJ mol^−1^), whereas in **4P**, the two hydrogen bonds have nearly identical strength (−30 kJ mol^−1^).

The next most attractive dimer interaction involves the aromatic rings, with comparable energies for interactions with methyl carbons (*sp*^3^)C—H⋯π in **BA** and **3P**, and interactions with aromatic carbons (*sp*^2^)C—H⋯π in **2P** and **4P**. In **4P**, four similar dimers with aromatic interactions exist and the one with the lowest energy marked with asterisk is presented in right-hand panel of Fig. 4 ▸. The weakest among calculated dimer interactions are methyl carbon with oxygen (*sp*^3^)C—H⋯O in **2P** and (*sp*^2^)C—H⋯π in **3P**. The three strongest dimer interactions described above determine the arrangement of molecules in crystal structures in a similar way in both even and odd alcohols. In the supporting information Hirshfeld surfaces calculated using *CrystalExplorer* (Spackman *et al.*, 2021 ▸) are presented. The maps show that strong O⋯H attractive contacts, representing hydrogen bonds, are located near the hydroxyl groups of alcohol molecules, while C⋯H contacts are found near aromatic rings and hydroxyl groups, indicating weaker aromatic interactions. However, the per cent contribution from aromatic interactions is twice that for hydrogen bonding. In general, hydrogen bonds are the strongest interactions within individual dimer contacts, driving the molecules to form a zigzag chain. On the other hand, aromatic interactions contribute the most to the crystal lattice energy, playing a more stabilizing role in the crystal structure.

### 2.4. Conformational and compressibility relationships within the phenyl series

In each system investigated, the number of molecular conformations is equal to the number of independent molecules *Z*′ (see Table 1 ▸), which is 2, 1, 1 and 2 for **BA**, **2P**, **3P** and **4P**, respectively. We did not observe any systematic changes of this value within the phenyl series. Instead, since each of these structures has a different space group, the number of conformations is related to the symmetry operations in each system. Analysis of the molecular conformations offers valuable insight into the organization of molecules within the crystal. The rigid aromatic ring has no possibility to bend, in contrast to other parts of molecule. On the contrary, the alkyl chain and hydroxyl group are easy to bend to adjust to intermolecular forces.

**Figure 5:**
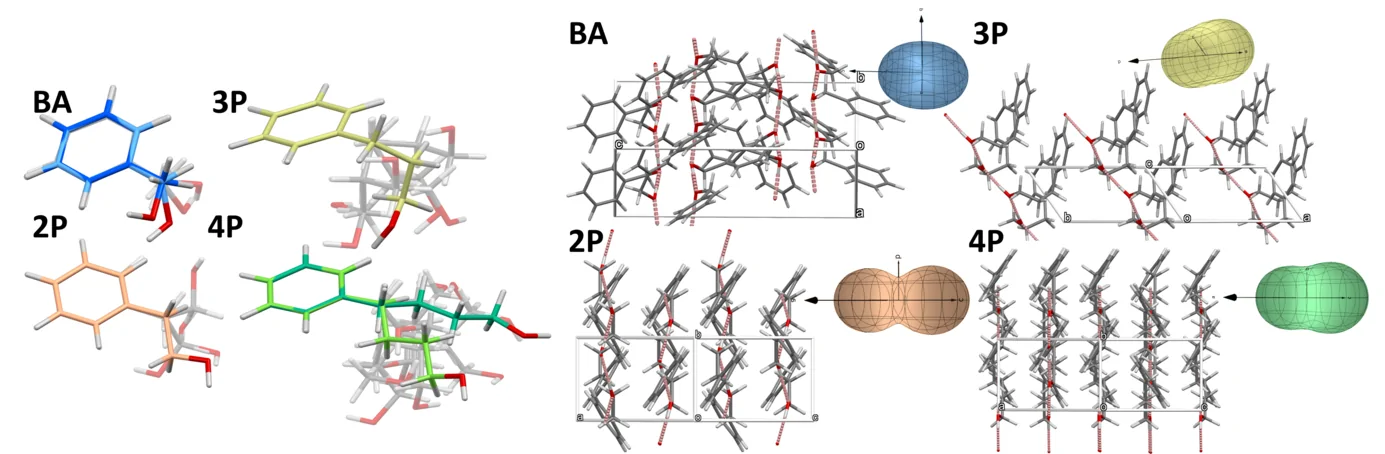
On the left, molecular conformations of the studied phenyl alcohols: DFT optimized at 0.8 GPa (colored molecules) and in the gas phase (gray molecules, see main text for references). On the right, the pressure compressibility of the optimized structures calculated for the studied phenyl alcohols over the pressure range 0.6–2.5 GPa. Axis directions match the presented crystal projections, and the units of compressibility are arbitrary, showing the relative compression in every axis direction.

All molecular conformations observed in the crystal structures were compared with gas-phase conformations available at *PubChem* (Kim *et al.*, 2013 ▸), and additionally, for **BA**, with the low-temperature crystal conformation (Nayak *et al.*, 2010 ▸). The results (Fig. 5 ▸) show overlapping molecular geometries, with high-pressure conformations highlighted. The number of possible conformations increases proportionally with molecular size. Notably, the conformations observed under high pressure are not predicted among the computed gas-phase conformers. The conformations found for **BA** are similar, only differing in the degree of hydroxyl group bending. For **2P**, a single conformer is observed in which the alkyl chain lies in the plane of the aromatic ring. In contrast, **3P** adopts a conformer with the alkyl chain bent almost perpendicular to the aromatic ring, likely caused by stress at high pressure. In **4P**, two conformers are present, both nearly planar with the aromatic ring, which look like mirror images of each other. A clear pattern is seen across the series of alcohols. In odd alcohols, the perpendicular hydroxyl group orientation enables the creation of (*sp*^3^)C—H⋯π interactions. In even alcohols, the long molecular axis aligned with the plane favors a layer arrangement.

In Fig. 4 ▸ experimental molecular volumes obtained from SR-SCXD and SR-PXD (open symbols) are compared with optimized ones (solid symbols). All alcohols exhibit positive pressure compressibility, with molecular volumes increasing with the molecular mass of the compound. The optimized volumes are systematically smaller than the experimental values by up to 5%, due to the absence of thermal expansion in the calculations. Structural optimizations performed at pressures of 0.6, 0.8, 1.3, 2.0 and 2.5 GPa were used for strain analysis in *PASCal* (Cliffe & Goodwin, 2012 ▸; Lertkiattrakul *et al.*, 2023 ▸). The resulting pressure compressibility tensors, visualized in Fig. 5 ▸, indicate that the directions of minimum compressibility align with the directions of hydrogen-bonded chains. In **BA**, where no clear separation between chains of hydrogen bonds is present, compressibility is more uniform in every direction. It is evident that hydrogen bonds, being the strongest interactions in the systems, govern the molecular organization under compression.

### 2.5. Similarities in structural organization ruled by alkyl chain length

Previous studies have documented odd–even effects in systems such as adducts of allylamine with alcohols (Prus *et al.*, 2022 ▸) or alkanes (Boese *et al.*, 1999 ▸; Dhiman *et al.*, 2022 ▸; Yang *et al.*, 2016 ▸). In short, the relatively higher melting points of even alkanes are explained as being a result of better packing than for odd members. Linear molecules of even alkanes have alternating up and down methyl groups, whereas for odd members all methyl groups are facing up or down. Therefore, less effective packing and a less dense system is observed for odd alkanes.

**Figure 6:**
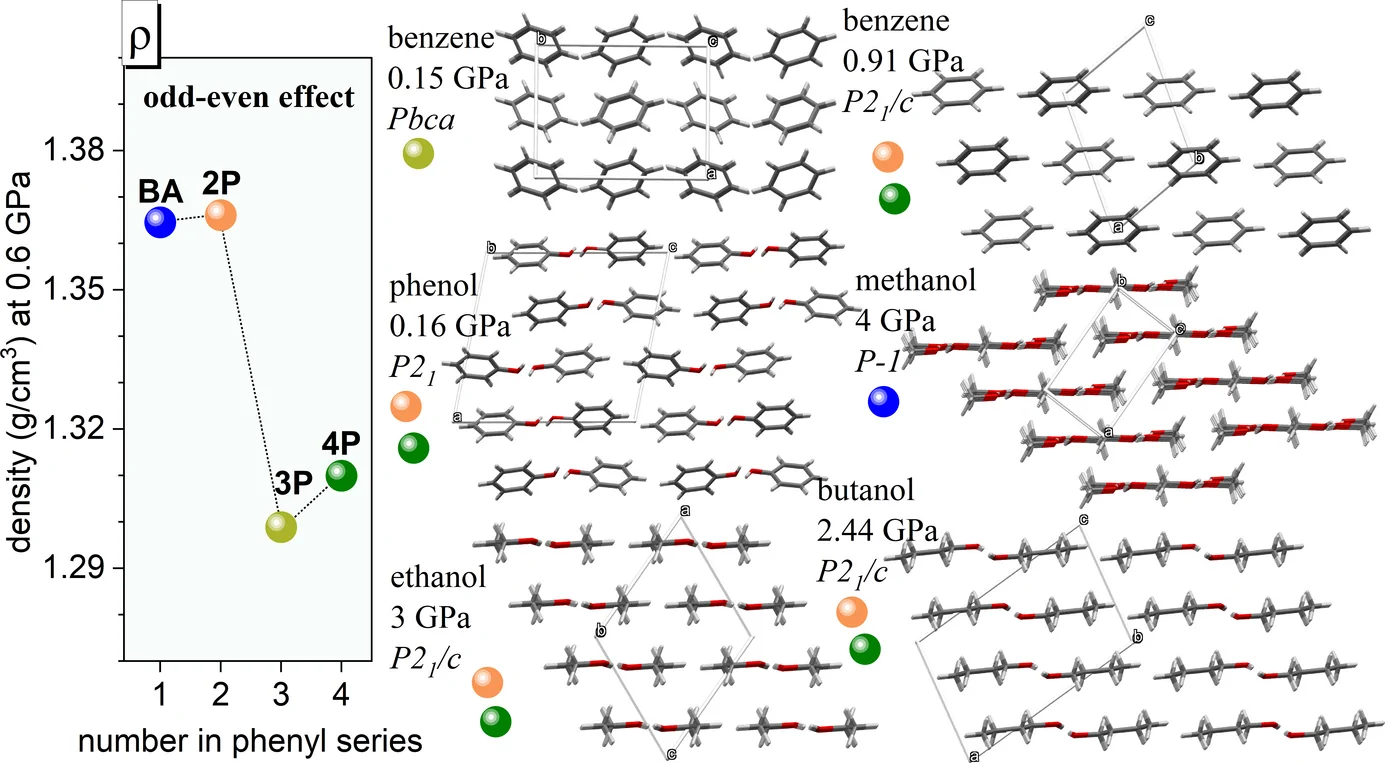
Densities calculated based on optimized systems at 0.6 GPa (left). Different high-pressure structures of alcohols and benzene (right), see main text for references. Literature structures that are similar to reported structures are marked with colored dots representing the investigated alcohol.

A similar odd–even effect is observed in the series of phenyl alcohols studied here. In Fig. 6 ▸ densities calculated at the same pressure point do not decrease monotonically with increasing molecular alkyl chain length. Instead, even compounds (**2P** and **4P**) exhibit higher densities than odd counterparts (**BA** and **3P**). This indicates more efficient molecular packing in even alcohols. In line with this observation, distinct features in structural organization are apparent between odd and even alcohols. In particular, **2P** and **4P** display closely related packing motifs of molecules organized within layers.

The high-pressure crystal structures reported here were compared with other published high-pressure structures. As illustrated in Fig. 6 ▸, the arrangement of aromatic rings in **3P** resembles that observed in benzene at 0.15 GPa (Katrusiak *et al.*, 2010 ▸), whereas the packing motifs in **2P** and **4P** are more similar to those found in benzene at 0.91 GPa (Katrusiak *et al.*, 2010 ▸). Furthermore, the structure of phenol at 0.16 GPa (Allan *et al.*, 2002 ▸), effectively an even alcohol, shows clear similarities in packing to those observed in **2P** and **4P**. Comparable motifs are also present in simple even alcohols, such as ethanol (Allan & Clark, 1999 ▸) (3 GPa) and *n*-butanol (Grelska *et al.*, 2025 ▸) (2.44 GPa), which also crystallize in related space groups, *P*2_1_/*c* or *P*2_1_. In contrast, the crystal structures of odd alcohols are not well defined. For example, the *n*-propanol crystal structure is not reported in the CSD, which could explain the delayed crystallization and lower density observed for **3P**. On the other hand, the odd alcohol methanol (Allan *et al.*, 1998 ▸) (4 GPa) shares a similar asymmetric molecular arrangement with its phenyl analog **BA**.

## 3. Conclusions

Four new crystal structures of phenyl alcohols series were determined under high pressure. Out of those, three alcohols from the series, **2P**, **3P** and **4P**, are the first known crystal structures of the pure compounds. All structures are characterized by the presence of hydrogen-bonded chains of molecules and stabilizing π-interactions. The crystallization was observed around 0.8 GPa for all the compounds, and structures at higher pressure points around 1.5 GPa were achieved for **BA**, **2P** and **3P**.

Energy calculations showed that these structures are not favorable above around 1.3 GPa. It seems that pressure is a key factor in inducing and maintaining crystallization of the phenyl alcohol series. Newly found structures have molecular conformations that have not been reported before. Among them, **BA** and **3P** have the hydroxyl group bent relative to the aromatic ring, which can favor strong hydrogen-bonding interactions. The **2P** and **4P** conformations are more planar and the face-to-face arrangement of molecules may result in better optimized van der Waals interactions and, as a consequence, slightly weaker hydrogen bonds. The calculations indicate that hydrogen bonding is the strongest dimer interaction in all alcohol structures and aromatic forces have the biggest area of interaction per molecule. In **BA** and **3P**, methyl carbon with aromatic ring interactions contribute to stabilization of the structures, whereas in **2P** and **4P** this role is played by interactions between the aromatic ring and aromatic protons.

Interestingly, the phenyl series structures exhibit an odd–even effect. The structures of **2P** and **4P** are denser and better packed compared with **BA** and **3P**, respectively. Moreover, the two even structures have an analogous packing motif, which is also common for other structures of even alcohols found in the literature. This study shows some common features in the organization of molecules in the crystal forms of structurally related alcohols of the phenyl series. It provides a benchmark for predicting and characterizing other high-pressure structures in the future.

## 4. Methods

### 4.1. High-pressure SR-PXD

Liquid samples of 2-phenylethanol and 4-phenyl-1-butanol were purchased from Sigma-Aldrich with a purity of more than 99.5%. High-pressure powder X-ray diffraction measurements of **4P** in a diamond anvil cell (DAC) were performed using the ID15B beamline at the European Synchrotron Radiation Facility (ESRF) in Grenoble, France. The incident beam wavelength was 0.411 Å. The preparation procedure and equipment parameters are analogous to those described in an earlier paper (Grelska *et al.*, 2025 ▸).

High-pressure powder X-ray diffraction measurements of **2P** in a DAC were carried out on the 16-BM-D beamline at the Advanced Photon Source, Argonne National Laboratory, USA. The incident beam wavelength was 0.619 Å. The preparation procedure and equipment parameters are analogous to those described in an earlier paper (Grelska *et al.*, 2024 ▸).

Both samples were placed as liquids in the DACs and pressurized using a membrane cell. Powder diffraction patterns were collected after crystallization occurred and also during decompression. Angular integration of two-dimensional diffraction patterns was performed using *Dioptas* (Prescher & Prakapenka, 2015 ▸) and analysis of powder diffraction data was performed using *Jana2020* software (Petříček *et al.*, 2023 ▸) in profile matching mode due to significant preferred orientation in the measured data.

### 4.2. High-pressure SCXD

Liquid samples of benzyl alcohol, 2-phenylethanol, 3-phenylpropanol and 4-phenyl-1-butanol were purchased from Sigma-Aldrich with a purity of more than 99.5%. All single-crystal experiments were performed using DACs built according to the Merrill and Bassett design and conducted at room temperature.

The procedure of crystallization was the same for all four alcohols. Firstly, liquid alcohol and some ruby chips (used to determine the pressure) were loaded into the DAC, and the pressure was manually increased to the point when crystallization to the polycrystalline form occurred. In the next step, the whole DAC was slowly heated using a heat gun. As the temperature inside the pressure chamber approached the melting point of the existing solid phase of the investigated alcohol, the polycrystalline form started melting. When there was only one unmelted crystallite left in the pressure chamber, the DAC was slowly cooled. Sometimes it was possible that only one single crystal grew until it filled the whole pressure chamber. Otherwise, a few single crystals grew in the chamber. After the sample reached room temperature, the pressure inside the DAC was checked by measuring the position of the fluorescence lines of ruby; the pressure uncertainty was 0.03 GPa. Crystal growth images taken under a microscope are shown in the supporting information.

Diffrcation data for samples prepared in this way were measured on an in-house diffractometer with a wavelength of 0.71073 Å. The *CrysAlis PRO* program (version 1.171.36.32, Agilent Technologies UK Ltd) was used for the data collection and their further reduction. The structures were solved by direct methods using *ShelXS* (Sheldrick, 2008 ▸) and refined with *ShelXL* (Sheldrick, 2015 ▸) within the *Olex2* suite (Dolomanov *et al.*, 2009 ▸). Owing to the better quality of data measured at the synchrotron sources for **BA**, **2P** and **3P**, only the data for **4P** measured on the in-house diffractometer are presented in Table 1 ▸ and in the supporting information.

### 4.3. High-pressure SR-SCXD

Additionally, data from the single crystals that were grown in DACs were measured using synchrotron radiation on beamline ID27 at the ESRF, Grenoble, France. The incident beam wavelength was 0.22292 Å. The samples of **BA**, **2P** and **3P** were measured at their crystallization points and at a manually increased pressure of around 1.5 GPa. Structural data for these compounds are presented in Table 1 ▸ and in the supporting information.

### 4.4. DFT calculations

In order to assess the relative stability of the crystal forms of the investigated alcohols with pressure and establish the hierarchy of intermolecular interactions, quantum-chemical calculations were carried out using the *CRYSTAL23* (Erba *et al.*, 2023 ▸) DFT code dedicated for periodic systems and utilizing Gaussian-orbital atomic basis sets. Calculations were performed using the B3LYP-D* method, in which B3LYP is augmented with an empirical dispersion term (Civalleri *et al.*, 2008 ▸; Grimme *et al.*, 2010 ▸), and Ahlrichs-VTZP basis sets (Peintinger *et al.*, 2013 ▸) dedicated for solid-state systems. TOLINTEG was set to 10 10 10 10 30 and SHRINK to 8 8.

Optimizations of the alcohol crystal structures were started from experimental high pressure SCXD geometries, measured around 0.8 GPa. The symmetry of each crystal structure was maintained during the optimization process. A full relaxation of both lattice parameters and atomic coordinates by means of analytical energy gradients was applied. In each case, a sequence of five periodic optimizations was performed with external pressures set to 0.6, 0.8, 1.3, 2.0 and 2.5 GPa. The resulting crystal structures were used for further lattice energy, dimer interaction energy and layer attachment energy calculations; see the details in the supporting information.

## Supplementary Material

Crystal structure: contains datablock(s) BA2_10GPa, BA0_68GPa, 4P0_4GPa, 3P1_33GPa, 3P0_66GPa, 2P1_21GPa, 2P0_72GPa. DOI: 10.1107/S2052252526009504/hay5001sup1.cif

Structure factors: contains datablock(s) BA_2_10GPa_corr. DOI: 10.1107/S2052252526009504/hay5001BA2_10GPasup2.hkl

Structure factors: contains datablock(s) BA_0_68GPa_corr. DOI: 10.1107/S2052252526009504/hay5001BA0_68GPasup3.hkl

Structure factors: contains datablock(s) 4P_0_4GPa_corr. DOI: 10.1107/S2052252526009504/hay50014P0_40GPasup4.hkl

Structure factors: contains datablock(s) 3P_1_33GPa. DOI: 10.1107/S2052252526009504/hay50013P1_33GPasup5.hkl

Structure factors: contains datablock(s) 3P_0_66GPa. DOI: 10.1107/S2052252526009504/hay50013P0_66GPasup6.hkl

Structure factors: contains datablock(s) 2P_1_21GPa. DOI: 10.1107/S2052252526009504/hay50012P1_21GPasup7.hkl

Structure factors: contains datablock(s) 2P_0_72GPa. DOI: 10.1107/S2052252526009504/hay50012P0_72GPasup8.hkl

Supporting information. DOI: 10.1107/S2052252526009504/hay5001sup9.pdf

Data from beamline ID15B: https://doi.org/10.15151/ESRF-ES-594732406

Data from beamline ID27: https://doi.org/10.15151/ESRF-ES-2248519405

CCDC references: 2587070, 2587071, 2587072, 2587073, 2587074, 2587075, 2587076

## Figures and Tables

**Table 1:** Basic crystallographic parameters of the studied phenyl alcohol series at 293 K and under various pressure conditions

	**BA**	**2P**	**3P**	**4P**
Space group	*P*2_1_2_1_2_1_	*P*2_1_/*c*	*Cc*	*P*2_1_
Pressure (GPa)	0.68	0.72	0.66	0.40
*a* (Å)	4.69	15.17	9.78	19.74
*b* (Å)	14.90	5.17	19.30	5.21
*c* (Å)	15.98	8.28	5.01	8.15
β (°)	90.00	96.87	128.64	91.90
Volume (Å^3^)	1115.5	644.9	738.8	838.5
*Z*/*Z*′	8/2	4/1	4/1	4/2
