# Supplementary material for: Noncovalent interactions and odd–even effect control the high-pressure crystal structures of phenyl alcohols
Source: IUCrJ. 2026 Nov 5;13(Pt 6):819–27. doi: 10.1107/S2052252526009504 (PMC13763889; doi:10.1107/S2052252526009504)
Supplement: Supplementary file 1 [file m-13-00819-sup9.pdf]

Supplementary Information for:

Noncovalent Interactions and Odd-Even Effect Control the High-Pressure Crystal Structures of Phenyl Alcohols

Joanna Grelska,[*,†] Roman Gajda,[†] Joanna Krzeszczakowska,[†]
Changyong Park,[‡] Krzysztof Woźniak,[†] and Wojciech A. Sławiński[*,†]

†*University of Warsaw, Faculty of Chemistry, Pasteura 1, 02-093 Warsaw, Poland*

‡*High Pressure Collaborative Access Team (HPCAT), X-ray Science Division, Argonne National Laboratory, Lemont, Illinois 60439, United States*

E-mail: j.grelska@uw.edu.pl; wslawinski@chem.uw.edu.pl

DFT analysis

Lattice energy was calculated according to the equation:

$$E_{lattice} = \frac{E_{total}}{Z} - E_{molecule} \tag{1}$$

where, Z is number of molecules and molecular energy was calculated using MOLECULE and MOLEBSSE keywords.

Interactions energies were calculated between pairs of molecules (see Table 2 for explanation how particular dimer was defined), according to the equation:

$$E_{interaction} = E_{dimmerAB} - (E_{moleculeA} + E_{moleculeB}) \tag{2}$$

Attachment energies of a two-molecule wide (100) layers were calculated according to the equation:

$$E_{attachment} = \frac{E_{total}}{2} - \frac{E_{SLAB}}{2} \tag{3}$$

The slabs have only 2D translational symmetry, and are two-molecules wide in (100) direction and infinite in (010) and (001) directions, see Fig. 1.

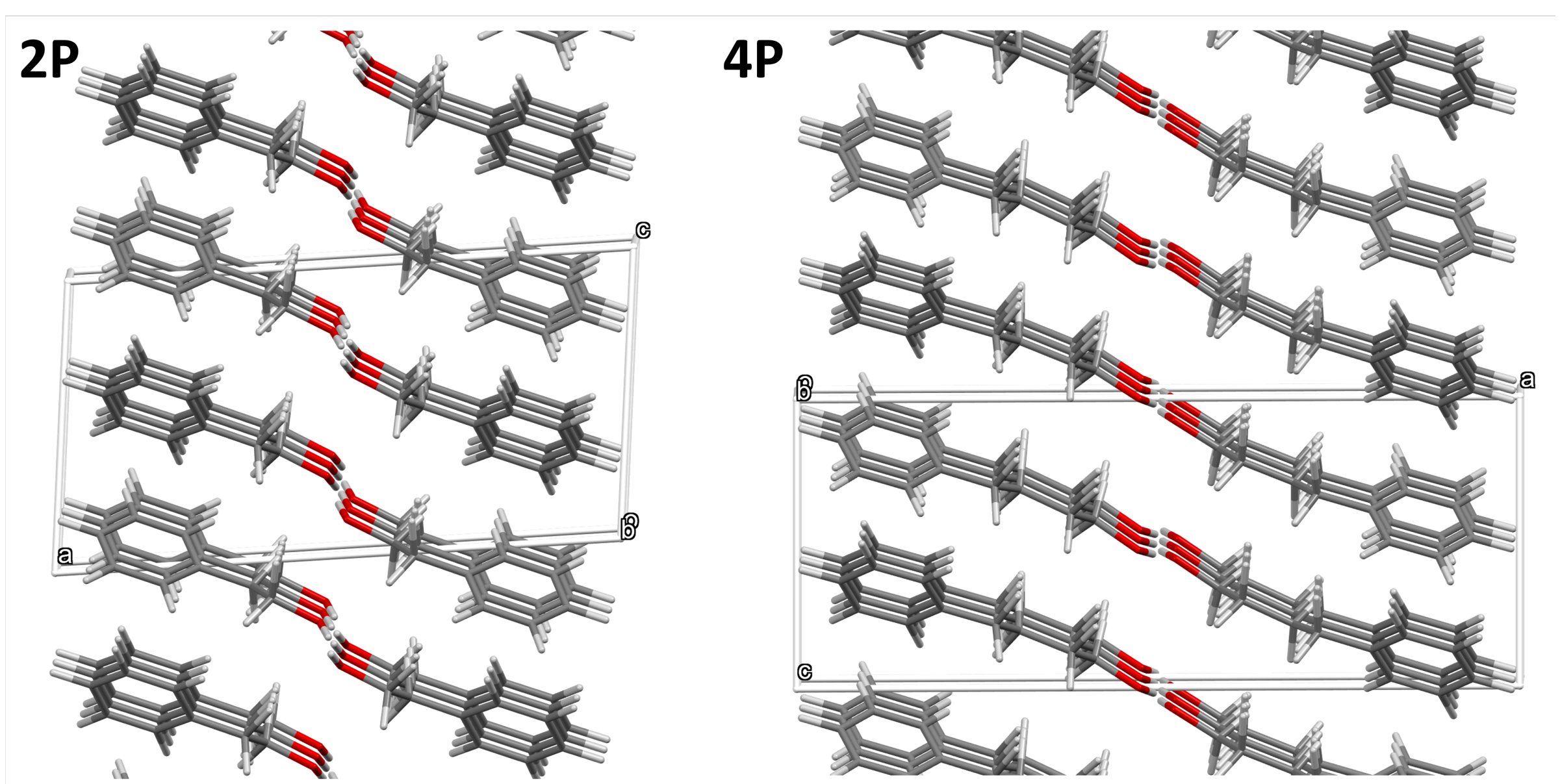

Figure 1: Slabs used in calculations of **2P** and **4P** systems.

Table 1: DFT calculated optimized structures and experimental Le Bail fitted *LB* unit cell parameters a, b, c, β angle, unit cell volume, lattice energy per molecule and layer energy per molecule *LE* at different pressure points. For some pressure points energy optimization of system could not be achieved.

**Pressure** (GPa)	**a** (Å)	**b** (Å)	**c** (Å)	β **angle** (°)	**Volume** (Å^3)	**Energy** (kJ/mol)
**BA**						
0.6	4.548864(6)	14.887769(6)	15.545292(6)	90.0	1052.7651(4)	-63.31518
0.8	4.542152(6)	14.787143(6)	15.551731(6)	90.0	1044.539123(6)	-51.27826
1.3	4.503015(6)	14.717098(6)	15.379995(6)	90.0	1019.252566(6)	-12.86835
2.0	4.446732(6)	14.711256(6)	15.123719(6)	90.0	989.348489(6)	39.32221
2.5	4.41121(5)	14.657793(6)	15.031164(6)	90.0	971.894069(6)	75.78232
**2P**						
0.6 *LE*						43.58651
0.6	15.147394(6)	5.055556(6)	7.800123(6)	96.056788	593.987345(6)	-61.61012
0.8	15.125526(6)	5.04824(5)	7.756647(6)	95.975207	589.058723(6)	-48.07264
1.3	15.062777(6)	5.024614(6)	7.634261(6)	95.769183	574.86965(5)	-4.59228
2.0	14.987503(6)	4.992516(6)	7.506772(6)	95.525708	559.086714(6)	54.77536
2.5	14.93651(5)	4.971172(6)	7.432198(6)	95.348194	549.452875(6)	96.12383
1.1 *LB*	15.16876	5.13802	8.15756	97.5101	630.3	
**3P**						
0.6	9.548013(6)	18.853319(6)	4.909198(6)	127.991622	696.45505(5)	-51.20244
0.8	9.516941(6)	18.79849(5)	4.896777(6)	127.989884	690.434843(6)	-35.41138
1.3	9.340158(6)	18.665754(6)	4.864066(6)	127.74759	670.531824(6)	17.02458
2.0	9.31325(5)	18.469844(6)	4.823459(6)	128.013843	653.692109(6)	84.3803
2.5	9.245641(6)	18.380589(6)	4.805741(6)	128.208908	641.722277(6)	132.57407
**4P**						
0.6 *LE*						59.98501
0.6	19.394134(6)	5.051826(6)	7.774284(6)	90.278956	761.682555(6)	-60.6753
0.8	19.34662(5)	5.041027(6)	7.745094(6)	90.15713	755.351733(6)	-43.30156
1.3	19.340217(6)	5.005949(6)	7.616658(6)	90.324283	737.403684(6)	12.59726
2.0	19.124756(6)	4.96786(5)	7.543483(6)	89.672315	716.687906(6)	—
2.5	18.900757(6)	4.944048(6)	7.546321(6)	88.652512	704.980358(6)	141.71529
1.0 *LB*	19.75951	5.17754	8.01566	91.65014	819.7	

Table 2: DFT calculated three lowest dimer energies E of optimized structures in pressure range. Corresponding interaction was assigned with distance d and angle θ choose as shortest length between intermolecular atoms (for Π being centroid of aromatic ring) and angle defined by atom order for O-H$\cdots$O, for C-H$\cdots\Pi$ defined as H$\cdots\Pi$-x (x being carbon atom connecting aromatic ring and alkyl chain) and for C-H$\cdots$O defined as H$\cdots$O-H. The asterisk in (sp^2)C-H$\cdots\Pi$* symbolizes the lowest energy dimer out of four equivalents.

**Pressure** (GPa)	E_1 (kJ/mol)	d_1 (Å)	θ_1 (°)	E_2 (kJ/mol)	d_2 (Å)	θ_2 (°)	E_3 (kJ/mol)	d_3 (Å)	θ_3 (°)
**BA**	**O-H$\cdots$O'**			**O'-H'$\cdots$O**			**(sp^3)C-H$\cdots\Pi$**		
0.6	-37.81877	1.641	178.17	-27.6794	1.653	174.15	-23.63968	2.743	79.59
0.8	-37.81101	1.636	177.83	-27.22417	1.648	173.44	-23.73598	2.71	80.12
1.3	-36.54331	1.621	177.97	-26.33775	1.632	173.09	-23.68005	2.723	79.09
2.0	-34.22555	1.606	178.09	-25.34431	1.613	173.16	-23.46325	2.774	77.01
2.5	-32.87893	1.593	178	-24.31446	1.599	172.75	-23.28254	2.771	76.33
**2P**	**O-H$\cdots$O**			**(sp^2)C-H$\cdots\Pi$**			**(sp^3)C-H$\cdots$O**		
0.6	-30.5187	1.817	177.06	-23.69104	2.637	88.48	-14.72956	2.543	112.07
0.8	-30.47463	1.813	177.1	-23.51305	2.622	88.33	-14.77709	2.532	112.33
1.3	-30.29214	1.799	177.18	-22.77829	2.582	87.85	-14.8817	2.5	113
2.0	-30.00375	1.782	177.27	-21.47539	2.537	87.37	-14.97312	2.462	113.73
2.5	-29.76802	1.77	177.27	-20.43525	2.509	87.14	-14.99384	2.436	114.13
**3P**	**O-H$\cdots$O**			**(sp^3)C-H$\cdots\Pi$**			**(sp^2)C-H$\cdots\Pi$**		
0.6	-40.48914	1.708	175.96	-22.1886	2.511	94.35	-15.50178	3.568	106.4
0.8	-40.26453	1.701	176.13	-22.0249	2.499	94.24	-15.39016	3.558	106.22
1.3	-40.46753	1.71	174.6	-16.93164	2.524	91.21	-14.43531	3.687	107.03
2.0	-38.12245	1.66	177.08	-20.29303	2.425	93.73	-14.13539	3.503	104.95
2.5	-37.50016	1.653	177.07	-19.01179	2.421	92.97	-13.34485	3.527	104.46
**4P**	**O-H$\cdots$O**			**O'-H'$\cdots$O'**			**(sp^2)C-H$\cdots\Pi$***		
0.6	-29.02029	1.762	176.26	-28.93732	1.763	176.13	-20.97396	2.638	94.25
0.8	-28.87524	1.755	176.29	-28.76439	1.756	176.45	-20.86294	2.623	94.29
1.3	-28.38327	1.734	176.51	-28.28012	1.736	176.35	-19.97217	2.58	95.05
2.0	-27.51335	1.706	176.88	-27.36458	1.709	177.29	-18.96266	2.534	94.72
2.5	-26.83926	1.688	177.04	-26.57878	1.691	177.92	-18.53887	2.51	93.35

PXD data

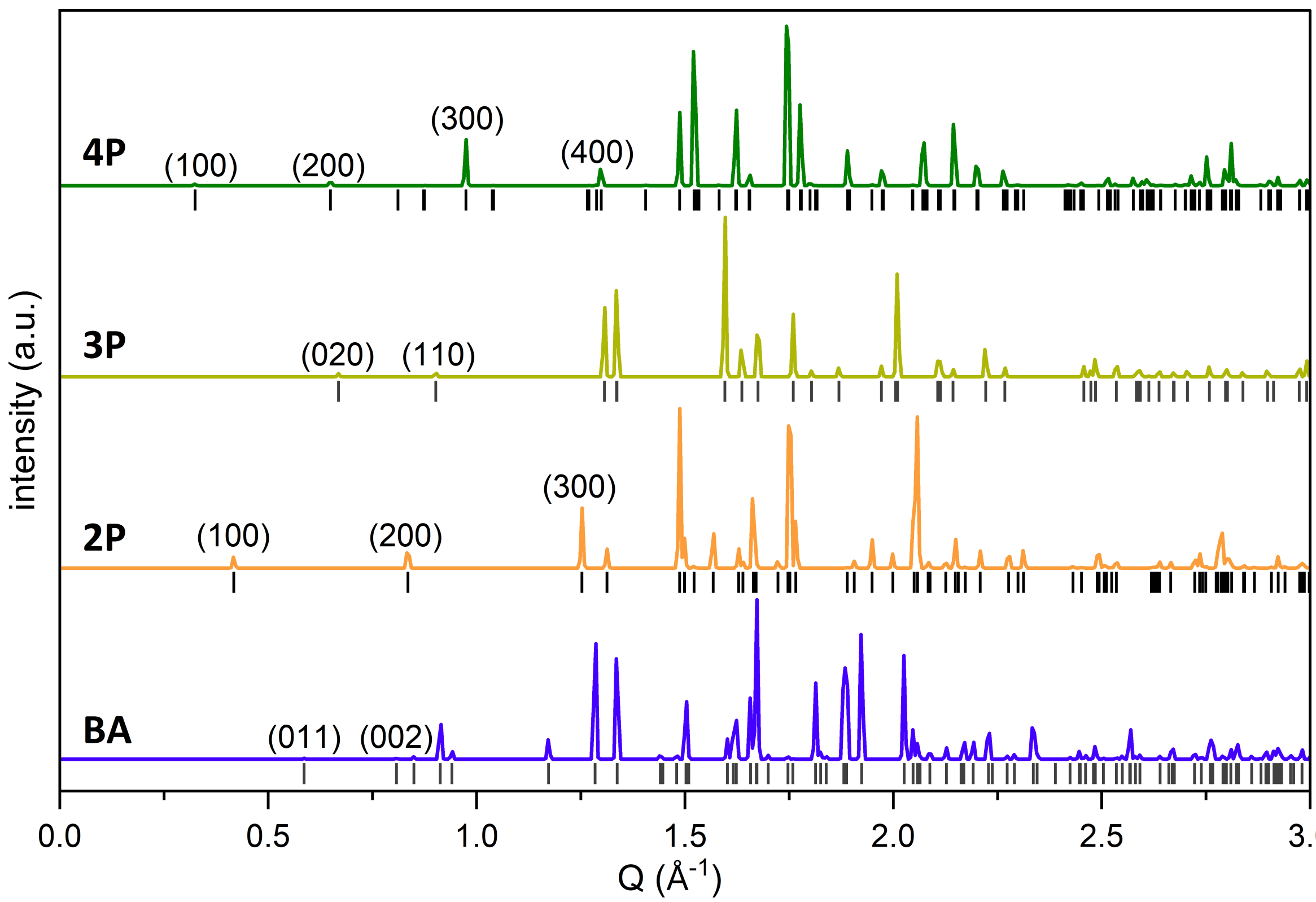

Figure 2: Simulated powder diffraction patterns of optimized at 0.8 GPa structures of **BA**, **2P**, **3P** and **4P**.

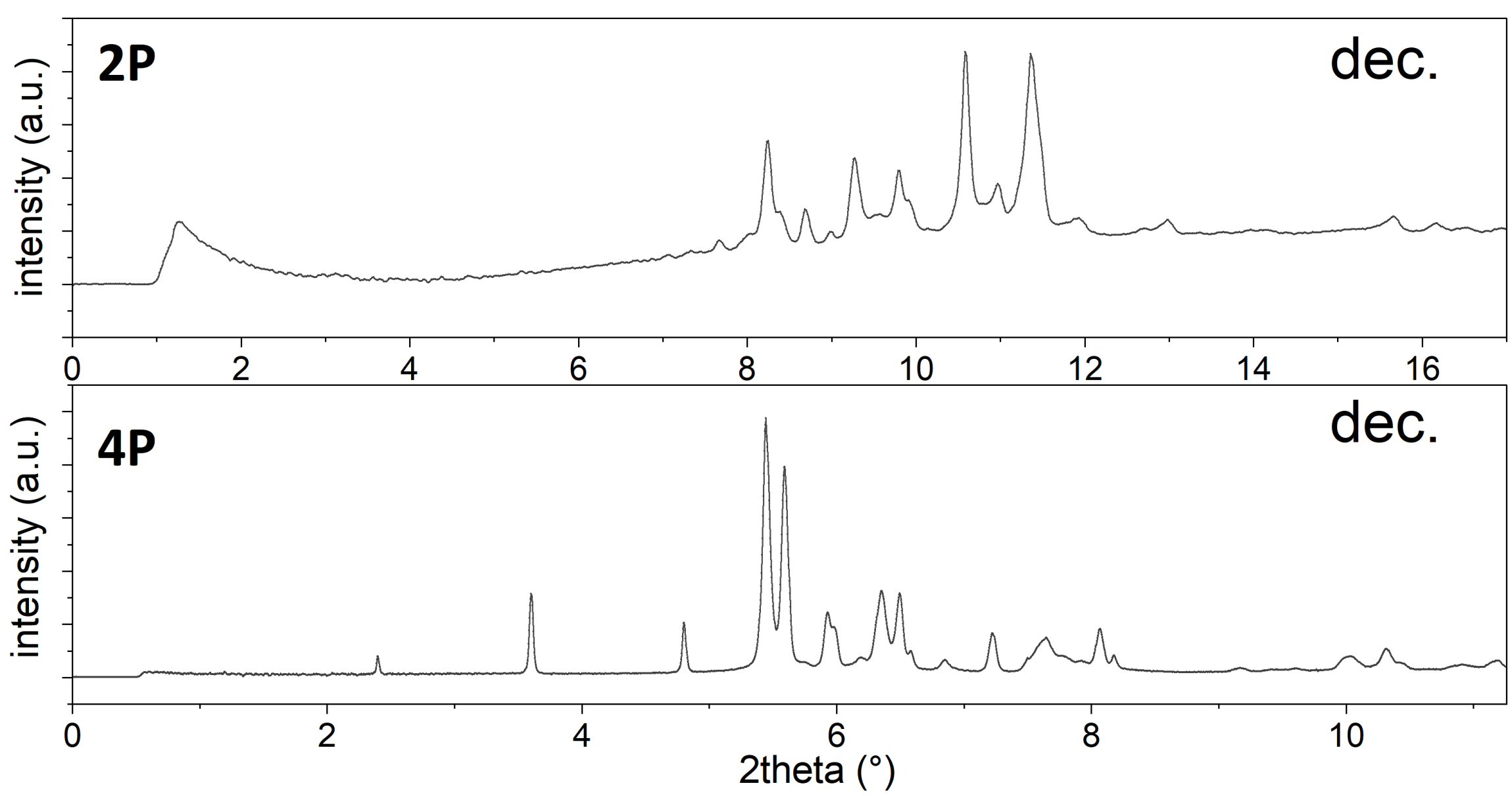

Figure 3: Experimental powder diffraction patterns measured on decompression of **2P** and **4P**.

SCXD samples

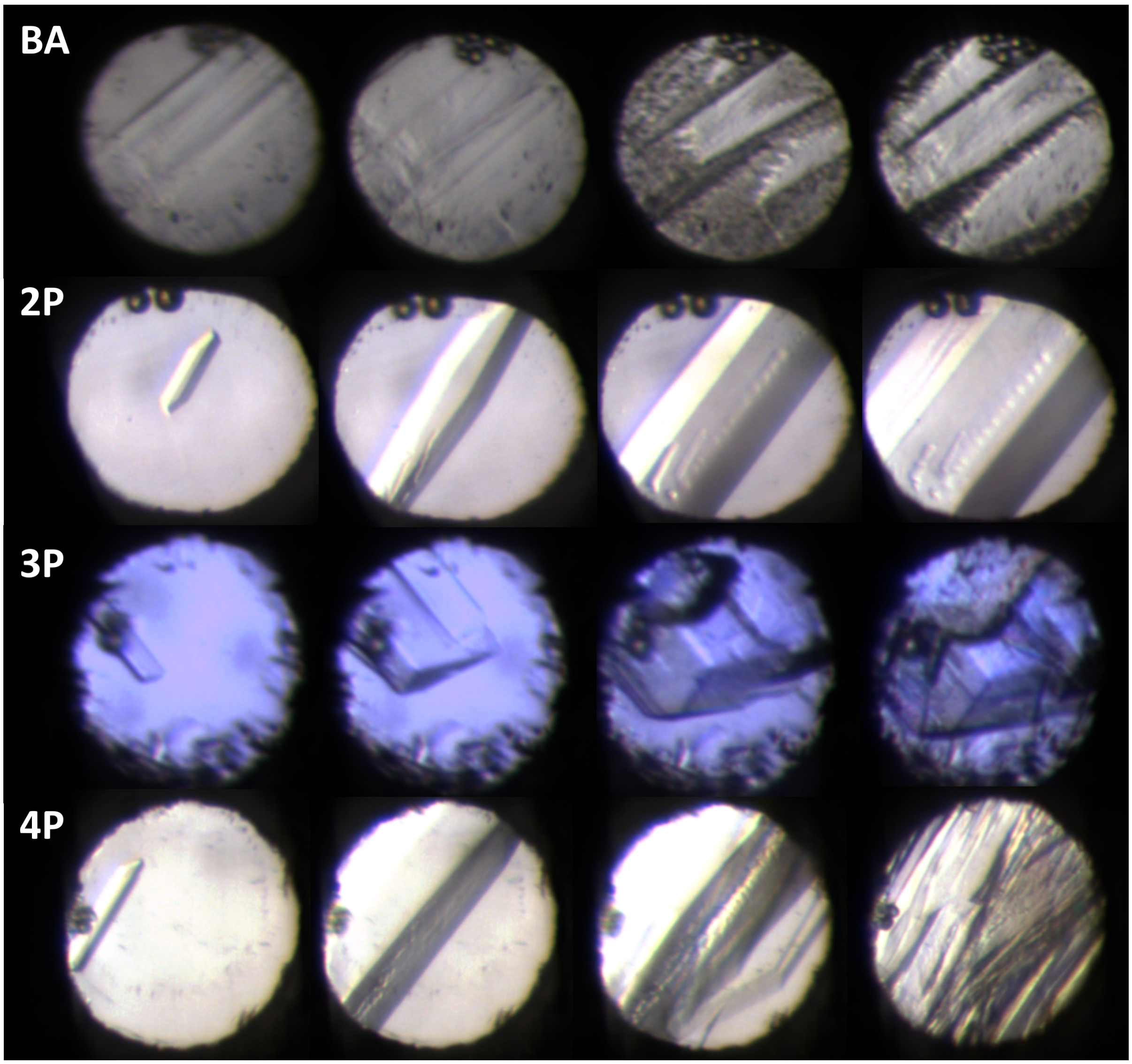

Figure 4: Crystals grown in diamond anvil cells under microscope for **BA**, **2P**, **3P** and **4P**.

Table 3: Selected refinement parameters and data collection details for measurements of **BA**.

Pressure (GPa)	0.68	2.10
Chemical formula	C_7H_8O	C_7H_8O
Molecular mass (g/mol)	108.13	108.13
Crystal system	orthorhombic	orthorhombic
Space group	$P2_12_12_1$	$P2_12_12_1$
a (Å)	4.6857 (3)	4.6217 (4)
b (Å)	14.9017 (12)	14.7169 (19)
c (Å)	15.975 (9)	15.447 (9)
Volume (Å^3)	1115.5 (6)	1050.6 (7)
Z	8	8
F(000)	465	464
D_x (Mg m^{-3})	1.291	1.368
Wavelength	0.22292	0.22292
No. of reflections for cell meas.	875	1043
θ range (°) for cell meas.	2.2-17.2	1.6-18.7
μ (mm^{-1})	0.03	0.03
Scan method	ω scans	ω scans
Absorption correction	multi-scan	multi-scan
T_{min}, T_{max}	0.012, 1,000	0.101, 1.000
Measured, independent, observed refl.	18449, 10138, 2229	18217, 9587, 2129
R_{int}	0.036	0.053
θ_{max}, θ_{min}	21.7, 1.6	21.7, 1.5
$(\sin\theta/\lambda)_{max}$ (Å^{-1})	1.661	1.661
Range of h, k, l	-13→15, -48→43, -18→15	-11→12, -34→41, -20→16
Refinement on	F^2	F^2
R [$F^2 > 2\sigma(F^2)$], wR(F^2), S	0.056, 0.197, 0.85	0.073, 0.267, 0.88
No. of reflections	10138	9587
No. of parameters	209	209
No. of restraints	0	14
$(\Delta/\sigma)_{max}$	<0.001	<0.001
$\Delta\rho_{max}$, $\Delta\rho_{min}$ (eÅ^{-3})	0.11, -0.10	0.18, -0.17

Table 4: Selected refinement parameters and data collection details for measurements of **2P**.

Pressure (GPa)	0.72	1.21
Chemical formula	$C_8H_{10}O$	$C_8H_{10}O$
Molecular mass (g/mol)	122.16	122.16
Crystal system	monoclinic	monoclinic
Space group	$P2_1/c$	$P2_1/c$
a (Å)	15.173 (2)	15.1840 (15)
b (Å)	5.1681 (3)	5.1210 (3)
c (Å)	8.2836 (17)	8.0258 (11)
β angle (°)	96.870 (16)	96.776 (13)
Volume (Å^3)	644.92 (16)	619.71 (11)
Z	4	4
F(000)	264	264
D_x (Mg m^{-3})	1.258	1.309
Wavelength (Å)	0.22292	0.22292
No. of reflections for cell meas.	560	661
θ range (°) for cell meas.	1.7-14.7	2.0–17.4
μ (mm^{-1})	0.02	0.03
Scan method	ω scans	ω scans
Absorption correction	multi-scan	multi-scan
T_{min}, T_{max}	0.82397, 1.000	0.741, 1.000
Measured, independent, observed refl.	12634, 7765, 1505	11224, 6940, 1770
R_{int}	0.061	0.034
θ_{max}, θ_{min}	21.9, 1.5	22.0, 1.5
$(\sin\theta/\lambda)_{max}$ (Å^{-1})	1.676	1.678
Range of h, k, l	-36→36, -14→15, -16→20	-40→39, -14→16, -16→17
Refinement on	F^2	F^2
R [$F^2 > 2\sigma(F^2)$], wR(F^2), S	0.096, 0.446, 0.88	0.093, 0.352, 0.92
No. of reflections	7765	6940
No. of parameters	122	122
No. of restraints	9	9
$(\Delta/\sigma)_{max}$	$<$0.001	$<$0.001
$\Delta\rho_{max}$, $\Delta\rho_{min}$ (eÅ^{-3})	0.23, -0.20	0.54, -0.23

Table 5: Selected refinement parameters and data collection details for measurements of **3P**.

Pressure (GPa)	0.66	1.33
Chemical formula	$C_9H_{12}O$	$C_9H_{12}O$
Molecular mass (g/mol)	136.19	136.19
Crystal system	monoclinic	monoclinic
Space group	Cc	Cc
a (Å)	9.7832 (15)	9.612 (3)
b (Å)	19.299 (3)	19.020 (3)
c (Å)	5.0101 (6)	4.924 (2)
β angle (°)	128.64 (2)	128.48 (5)
Volume (Å^3)	738.8 (2)	704.7 (6)
Z	4	4
F(000)	296	296
D_x (Mg m^{-3})	1.224	1.284
Wavelength (Å)	0.22292	0.22292
No. of reflections for cell meas.	539	473
θ range (°) for cell meas.	1.9–15.0	2.0–15.4
μ (mm^{-1})	0.02	0.03
Scan method	ω scans	ω scans
Absorption correction	multi-scan	multi-scan
T_{min}, T_{max}	0.354, 1.000	0.528, 1.000
Measured, independent, observed refl.	7469, 6233, 1723	6876, 5926, 1804
R_{int}	0.032	0.053
θ_{max}, θ_{min}	21.8, 1.6	21.9, 1.7
$(\sin\theta/\lambda)_{max}$ (Å^{-1})	1.664	1.671
Range of h, k, l	-26→28, -34→43, -15→14	-20→20, -57→55, -7→10
Refinement on	F^2	F^2
R [$F^2 > 2\sigma(F^2)$], wR(F^2), S	0.062, 0.237, 0.95	0.075, 0.286, 0.91
No. of reflections	6233	5926
No. of parameters	95	95
No. of restraints	2	2
$(\Delta/\sigma)_{max}$	<0.001	<0.001
$\Delta\rho_{max}$, $\Delta\rho_{min}$ (eÅ^{-3})	0.13, -0.12	0.22, -0.15

Table 6: Selected refinement parameters and data collection details for measurements of **4P**.

Pressure (GPa)	0.40
Chemical formula	$C_{10}H_{14}O$
Molecular mass (g/mol)	150.22
Crystal system	monoclinic
Space group	$P2_1$
a (Å)	19.741 (14)
b (Å)	5.2122 (4)
c (Å)	8.154 (2)
β angle (°)	91.90 (5)
Volume (Å^3)	838.5 (7)
Z	4
F(000)	328
D_x (Mg m^{-3})	1.190
Wavelength (Å)	0.71073
No. of reflections for cell meas.	1466
θ range (°) for cell meas.	4.0–27.3
μ (mm^{-1})	0.08
Scan method	ω scans
Absorption correction	Multi-scan
T_{min}, T_{max}	0.500, 1.000
Measured, independent, observed refl.	3327, 1504, 888
R_{int}	0.048
θ_{max}, θ_{min}	31.2, 2.7
$(\sin\theta/\lambda)_{max}$ (Å^{-1})	0.728
Range of h, k, l	-17→16, -7→7, -9→10
Refinement on	F^2
R [$F^2 > 2\sigma(F^2)$], wR(F^2), S	0.129, 0.383, 1.24
No. of reflections	1504
No. of parameters	91
No. of restraints	43
$(\Delta/\sigma)_{max}$	<0.001
$\Delta\rho_{max}$, $\Delta\rho_{min}$ (eÅ^{-3})	0.54, -0.27

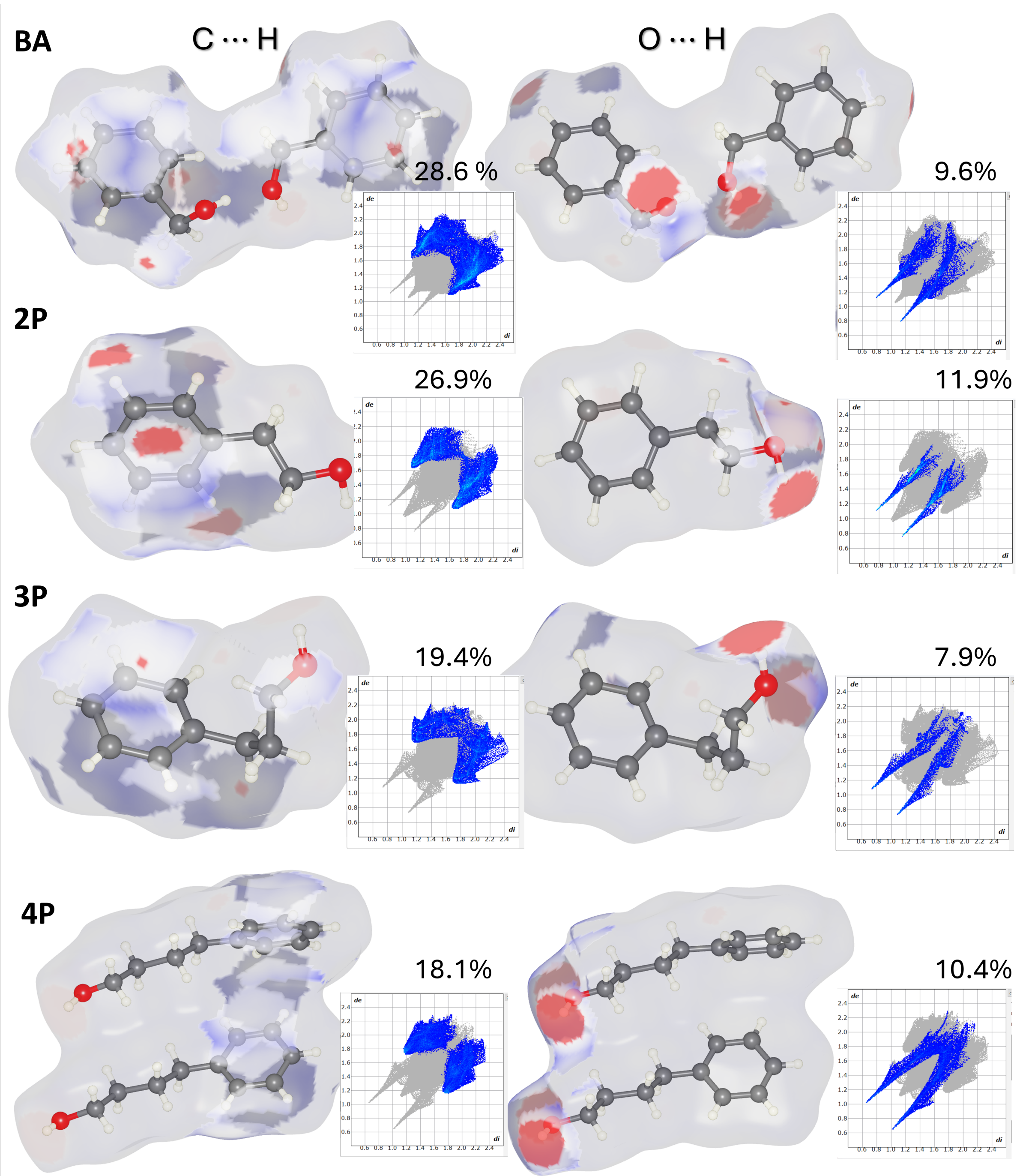

Figure 5: Hirshfeld surfaces for lower pressure experimental structures of **BA**, **2P**, **3P** and **4P**.
